# Occurrence, serotypes and virulence characteristics of Shiga toxin-producing and Enteropathogenic *Escherichia coli* isolates from dairy cattle in South Africa

**DOI:** 10.1007/s11274-024-04104-w

**Published:** 2024-08-13

**Authors:** Alaba S. Olawole, Mogaugedi N. Malahlela, Thierry Y. Fonkui, Munyaradzi C. Marufu, Beniamino T. Cenci-Goga, Luca Grispoldi, Eric M. C. Etter, Whatmore M. Tagwireyi, Musafiri Karama

**Affiliations:** 1 Department of Paraclinical Sciences, Faculty of Veterinary Science, Veterinary Public Health Section, University of Pretoria, Onderstepoort, 0110 South Africa; 2 Department of Veterinary Tropical Diseases, Faculty of Veterinary Science, University of Pretoria, Onderstepoort, 0110 South Africa; 3 Departiment of Veterinary Medicine, Laboratorio Di Ispezione Degli Alimenti Di Origine Animale, University of Perugia, 06126 Perugia, Italy; 4 CIRAD, UMR ASTRE, 97170 Petit-Bourg, Guadeloupe France; 5 ASTRE, University de Montpellier, CIRAD, INRAE, 34398 Montpellier, France; 6 Clinical Sciences, School of Veterinary Medicine, Ross University, P.O. Box 334, Basseterre, West Indies St Kitts and Nevis

**Keywords:** Dairy cattle, EPEC, STEC, Serotypes, Virulence, South Africa

## Abstract

Shiga toxin-producing and Enteropathogenic *Escherichia* *coli* are foodborne pathogens commonly associated with diarrheal disease in humans. This study investigated the presence of STEC and EPEC in 771 dairy cattle fecal samples which were collected from 5 abattoirs and 9 dairy farms in South Africa. STEC and EPEC were detected, isolated and identified using culture and PCR. Furthermore, 339 STEC and 136 EPEC isolates were characterized by serotype and major virulence genes including *stx1*, *stx2*, *eaeA* and *hlyA* and the presence of *eaeA* and *bfpA* in EPEC. PCR screening of bacterial sweeps which were grown from fecal samples revealed that 42.2% and 23.3% were STEC and EPEC positive, respectively. PCR serotyping of 339 STEC and 136 EPEC isolates revealed 53 different STEC and 19 EPEC serotypes, respectively. The three most frequent STEC serotypes were O82:H8, OgX18:H2, and O157:H7. Only 10% of the isolates were classified as “Top 7” STEC serotypes: O26:H2, 0.3%; O26:H11, 3.2%; O103:H8, 0.6%; and O157:H7, 5.9%. The three most frequent EPEC serotypes were O10:H2, OgN9:H28, and O26:H11. The distribution of major virulence genes among the 339 STEC isolates was as follows: *stx1*, 72.9%; *stx2*, 85.7%; *eaeA*, 13.6% and *hlyA,* 69.9%. All the 136 EPEC isolates were *eaeA*-positive but *bfp*A*-*negative, while 46.5% carried *hlyA.* This study revealed that dairy cattle are a major reservoir of STEC and EPEC in South Africa. Further comparative studies of cattle and human STEC and EPEC isolates will be needed to determine the role played by dairy cattle STEC and EPEC in the occurrence of foodborne disease in humans.Please kindly check and confirm the country and city name in affiliation [6].This affiliation is correct.Please kindly check and confirm the affiliationsConfirmed. All Affiliations are accurate

**Supplementary Information:**

The online version contains supplementary material available at 10.1007/s11274-024-04104-w.

## Introduction

Shiga toxin-producing *Escherichia coli* (STEC) and Enteropathogenic *Escherichia coli* (EPEC) are enteric pathogens commonly associated with diarrheal disease in humans. STEC disease is characterized by mild to severe diarrhea and complications such as hemorrhagic colitis (HC) and hemolytic uremic syndrome (HUS). EPEC is one of the leading causes of acute secretory watery diarrhea in children less than two years of age, worldwide, particularly in developing countries (GDB 2018).

Ruminants including cattle are the main animal reservoirs of STEC and EPEC (Habets et al. 2020; Hussein & Sakuma 2005a, b; Singh et al. 2015). Both STEC and EPEC are shed in the feces of cattle which may end up contaminating food products, water and the environment (Farrokh et al. 2013; Kintz et al. 2017). The occurrence of STEC and EPEC in cattle can be influenced by a number of factors, including the age of the animal, diet type, season and management practices (Cray et al. 1998; Gunn et al. 2007; Venegas-Vargas et al. 2016). Humans can acquire STEC and/or EPEC infections by ingesting contaminated beef or dairy products, vegetables or water (Hernandes et al. 2009; Pires et al. 2019). Furthermore, both STEC and EPEC can be transmitted to humans through contact with infected animals and a contaminated environment or from person to person (Hernandes et al. 2009; Kintz et al. 2017).

The main virulence factors of STEC are bacteriophage-encoded Shiga toxins (Stx1 and Stx2) (O'Brien et al. 1983; Strockbine et al. 1986). Another major virulence factor of STEC and EPEC is intimin (*eaeA*) (Jerse et al. 1990; McDaniel et al. 1995; Tzipori et al. 1995). Intimin is an adhesin responsible for attachment of STEC and EPEC to enterocytes and formation of typical attaching and effacing lesions observed in the intestinal epithelium of humans or animal models infected with *eaeA*-positive STEC or EPEC strains (Jerse et al. 1990; McDaniel et al. 1995; Tzipori et al. 1995). Furthermore, STEC and EPEC may carry a plasmid-encoded hemolysin (*hlyA*) which has been associated with enhanced virulence and severe infection. (Schmidt & Karch 1996).

While STEC and EPEC may share identical virulence factors (*eaeA* and *hlyA*), EPEC lacks bacteriophage-encoded Shiga toxins. Specifically, some EPEC strains possess a bundle-forming pilus *(bfpA*) which is located on the EPEC adherence factor (EAF) virulence plasmid (Girón et al. 1991, 1993). Bfp is required for the typical localized adherence (LA) phenotype commonly observed in the intestinal epithelium of animal models experimentally infected with *bfp*-positive EPEC strains (Cleary et al. 2004; Tobe & Sasakawa 2001). EPEC strains that possess *bfpA* are “typical EPEC” (tEPEC), while *bfpA-*negative EPEC are termed “atypical EPEC” (aEPEC) (Kaper et al. 2004; Trabulsi et al. 2002). Humans are the main source and reservoir of typical EPEC strains, while humans and healthy and/or diseased animals are considered sources and reservoirs of atypical EPEC strains (Hernandes et al. 2009; Hu & Torres 2015).

Although STEC and EPEC have been previously detected in cattle and associated with human disease, worldwide (Alfinete et al. 2022; GDB 2018; Habets et al. 2020; Karama et al. 2019a; Mainga et al. 2018; Pires et al. 2019; Smith et al. 2019), studies on the occurrence and characteristics of STEC from dairy cattle in South Africa are scarce, while similar reports on EPEC are non-existent. Therefore, the objectives of this study were (i) to determine the occurrence of STEC and EPEC in dairy cattle in South Africa and (ii) characterize recovered STEC and EPEC isolates by serotype and major virulence-associated genes.

## Materials and methods

### Study population and sample collection

In this study, a total of 771 fecal samples were obtained from dairy cattle and screened for STEC and EPEC. Fecal samples were collected from all adult cattle which were present at the dairy farms and abattoirs surveyed on the day of sampling**.** Fecal samples were collected on 9 dairy cattle farms (n = 404) and spent dairy cattle at 5 abattoirs (n = 367) in 3 provinces (Gauteng, Eastern Cape and Free State) of South Africa. The 5 abattoirs and 9 farms are represented by letters (A–E) and (F–N), respectively. Fresh fecal samples were collected and transported on ice to the laboratory, where they were stored at 4 °C and processed within one week. This study was approved by the Faculty of Veterinary Science, University of Pretoria, Research Ethics and Animal Ethics Committees-under approval numbers REC033-23 and REC 109-19, respectively.

### Sample enrichment and culture

All fecal samples were enriched by placing 5 g of feces of each sample in 45 mL of EC broth (CM0990, Oxoid, Basingtoke, UK) containing 20 mg/L of novobiocin (N1628, Sigma Aldrich, St. Louis, MO, USA) and incubated at 37 °C in a shaking incubator for 18–24 h (Mainga et al. 2018). A 100 µL aliquot of the enriched culture was spread on Drigalski lactose agar (CM0531, Oxoid, Basingtoke, UK) and CHROMagar STEC base ST162(B) containing the ST162(S) supplement (CHROMagar™, Paris, France) and incubated at 37 °C for 18–24 h.

### DNA extraction, STEC and EPEC screening by PCR

DNA was extracted by the boiling method (Malahlela et al. 2022) from all Drigalski lactose agar and CHROMagar petri dishes that showed growth. Briefly, a loopful of bacterial colony sweep was obtained from each Petri dish and placed in an Eppendorf tube containing 1 mL of FA buffer (223,143, BD Difco, Sparks, MD, USA). The bacterial suspension was washed twice in FA buffer by vortexing and centrifugation (10800 × *g* for 5 min). The supernatant was discarded after each washing. The pellet formed after the 2nd washing cycle was resuspended in 500 µL of sterile water. The suspension was homogenized and boiled at 100 °C for 25 min in a dry heating block and cooled immediately on ice for 10 min. The lysate containing the DNA was stored at −20 °C for later use. To detect STEC and EPEC, DNA was thawed and centrifuged, and multiplex PCR (mPCR) was used to screen the DNA template for *stx*1, *stx*2, *eaeA* and *hlyA* as previously described (Paton & Paton 1998). The 25 µL PCR mixture consisted of 5 μL of supernatant DNA template, 2.5 μL of 10X Thermopol reaction buffer, 1 unit of Taq DNA polymerase, 2 μL of 2.5 mM dNTPs (deoxynucleotide triphosphates), 0.6 µL for each of the forward and reverse primers (10 μM final concentration) (Inqaba Biotec, Pretoria, South Africa) and 10.5 μL of sterile water. DNA from the STEC O157:H7 strain EDL933 (ATCC®43,895™) was used as the PCR positive control for STEC (*stx1*, *stx2*, *eaeA* and *hlyA*), and sterile water was used as the negative control. PCR products were electrophoresed in a 2% agarose gel in TAE buffer (Tris–acetate-ethylenediamine tetra acetic acid), stained in a solution of ethidium bromide and visualized under ultraviolet (UV) light in a Gel Doc™ imaging system XR^+^ (Bio‐Rad, Hercules, USA). All Drigalski lactose agar and CHROMagar sample plates which were positive on initial PCR screening for *stx1* and/or *stx2* were considered STEC positive; while those which were *stx*-negative but positive for *eaeA*, were considered EPEC-positive.

### Isolation and detection of STEC and EPEC isolates

Purified suspect STEC and EPEC single colonies were obtained by streaking colony sweeps from all STEC and/or EPEC PCR-positive Drigalski lactose agar and CHROMagar STEC plates onto fresh Drigalski lactose agar and CHROMagar plates. The plates were incubated at 37 °C for 18–24 h. Up to five single colonies were picked from each plate and individually propagated and multiplied on Luria Bertani (LB) agar (Becton and Dickinson & Company, Sparks, USA). Thereafter, DNA was extracted from each purified single colony by the boiling method as described above. PCR was performed as described above to confirm the STEC or EPEC status of each single colony (Paton & Paton 1998). All purified single colonies which were PCR-positive for *stx1* and/or *stx2* were confirmed as STEC, while those negative for *stx* but positive for *eaeA* were considered EPEC. Furthermore, an additional PCR (Gunzburg et al. 1995) was performed to classify EPEC into typical EPEC (*bfpA*-positive) and atypical EPEC (*bfpA-*negative) strains. Polymerase chain reaction was carried out to verify whether each confirmed STEC or EPEC pure colony was indeed *E. coli* using a PCR protocol and primers previously described by Doumith et al., (2012). Bacterial sweeps of purified single colonies that were confirmed as STEC or EPEC were collected from LB agar plates and preserved at −80 °C in cryovials containing a freezing mixture 70% Brain heart infusion broth (53,286, Sigma-Aldrich) and 30% glycerol until further processing.

### Molecular serotyping of STEC and EPEC isolates

STEC and EPEC isolates were serotyped (O:H) using previously described PCR protocols (Banjo et al. 2018; Iguchi et al. 2015, 2020; Singh et al. 2015). Previously serotyped STEC isolates in our collection (Karama et al. 2019b; Mainga et al. 2018; Malahlela et al. 2022) and additional STEC which had been previously serotyped at the Laboratorio de Referencia de *Escherichia coli* (LREC), Facultad de Veterinaria, Universidad de Santiago de Compostela, Lugo, Spain and the National Microbiology Laboratory, Public Health Agency of Canada, Guelph, Ontario, Canada, were used as positive controls for PCR serotyping. In addition, reference STEC strains which were kindly provided by the European Union Reference Laboratory for *Escherichia coli*, Istituto Superiore di Sanità, Rome Italy, were used as positive controls for serotypes belonging to the following “Top 7/Big 7” STEC serogroups: STEC-C210-03 (O157), STEC-ED476 (STEC O111), STEC-C1178-04 (STEC O145), STEC-C125-06 (STEC O103) and STEC-ED745 (O26).

### Statistical analysis

Data were summarized and described using proportions and percentages in Microsoft Excel spreadsheets. The Fisher’s exact test was used to determine whether there were statistical differences between the occurrence of STEC and EPEC among cattle on dairy farms and spent dairy cattle at abattoirs. A p value of 0.05 was considered statistically significant. A 2 × 2 contingency table was used to calculate the odds ratios for the occurrence of STEC and EPEC in dairy cattle on farms and at abattoirs. The convenient sampling approach was used to determine the sample size for this study. However, to adjust for the clustering effect (intra-cluster) in the cattle herds/farms surveyed, the 95% confidence interval (CI) was calculated by taking into account the cluster/farm size and assuming an intra-class correlation coefficient of 0.1 using the formulas by Dohoo et al. (2003). Statistical analysis was performed using Python Script software: https://www.python.org/about/gettingstarted/

## Results

### Occurrence of STEC in dairy cattle at abattoirs and farms

**Fig. 1:**
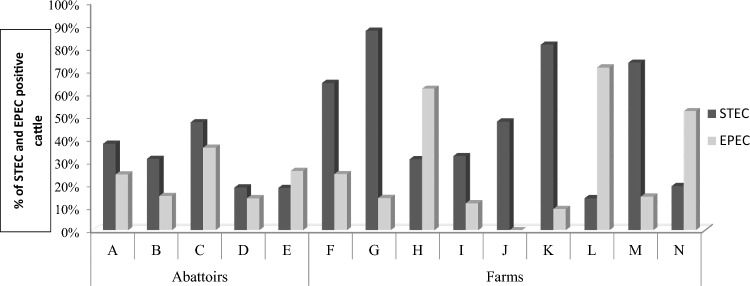
STEC and EPEC occurrence in dairy cattle at abattoirs and dairy farms

Overall, STEC was detected in 42.2% (325/771) (95% Confidence interval: 38.7–45.6%) of dairy cattle faeces. Furthermore, 29.9% (110/367) (95% CI: 25.3–34.7%) of spent dairy cattle faeces samples which were collected from abattoirs (A–E) and 53.2% (215/404) (95% CI: 48.4%–58.1%) from dairy cattle on farms (F–N) were STEC positive. There was a significantly lower likelihood (Odds ratio: 0.376; p < 0.05) of finding STEC in spent dairy cattle at abattoirs compared to dairy cattle on farms. The proportion of STEC positive cattle per abattoir (A–E) and farm (F–N) tested as follows: A, 37.8% (34/90); B, 31.2% (34/107); C, 47.2% (17/36); D, 18.7% (20/107); E, 18.5% (5/27) F, 64.6% (42/65); G, 87.5% (56/64); H, 31.0% (9/29); I, 32.4% (11/34); J, 47.5% (19/40); K, 81.4% (35/43); L, 14.3% (1/7); M, 73.5% (25/34); and N, 19.3% (17/88) (Table 1, Fig. 1).

**Table 1:** STEC occurrence and serotypes in dairy cattle at abattoirs and dairy farms

Abattoirs (A–E) farms (F–N)	Prevalence	O serogroups (N = 35)	Serotypes (N = 53)	Isolates (N = 339)	Dairy cattle (N = 771)
A (Number of cattle tested = 90)	(37.8%) 34/90	O8	O8:H8	2	1
		O8	**O8:H19**	1	1
		O27	O27:H21	1	1
		O92	O92:H28	1	1
		O153/O178	**O153/O178:H7**	1	1
			**O153/O178:H19**	3	1
			O153/O178:H21	1	1
		O163	O163:H21	1	1
		O174	**O174:H28**	1	1
		O182	**O182:H25**	5	1
		OgN3	OgN3:H2	18	3
		OgX25	OgX25:H11	1	1
B (Number of cattle tested = 107)	(31.2%) 34/117	O8 (8)	O8:H9	1	1
			O8:21	5	4
			O8:H28	1	1
			O8:H38	1	1
		O22	**O22:H16**	8	2
		O38	O38:H8	1	1
		O43	O43:H8	1	1
		O61	O61:H16	3	1
		O110	O110:H28	10	1
		O139	O139:H15	1	1
		O157	**O157:H7**	2	1
		O167	O167:H25	1	1
		OgN13	OgN13:H19	5	1
		OgX18	OgX18:H2	5	1
		ONT	ONT:H7	1	1
			ONT:H8	2	2
C (Number of cattle tested = 36)	(47.2%) 17/36	O8	**O8:H21**	4	1
		O136	O136:H16	1	1
		O153/O178	O153/O178:H19	7	1
			O153/O178:H49	1	1
		O157	**O157:H7**	4	1
D (Number of cattle tested = 107)	(18.7%) 20/107	O2/O50	O2/O50:H45	5	1
		O98	O98:H28	3	1
		OgN33	OgN33:H19	1	1
		OgX18	OgX18:H2	6	2
		OgX25	OgX25:H11	4	1
			OgX25:H28	1	1
		ONT	ONT:H39	1	1
E (Number of cattle tested = 27)	(18.5%) 5/27	O22	**O22:H8**	3	1
			**O22:H11**	2	1
		O103	**O103:H8**	2	1
		O110	**O110:H19**	2	1
		O153/O178	O153/O178:H19	1	1
		ONT	ONT:H19	2	1
F (Number of cattle tested = 65)	(64.6%) 42/65	O84	**O84:H2**	1	1
		O2/O50	O2/O50:H45	1	1
		O139	O139:H8	1	1
		O24	O24:H38	2	1
G (Number of cattle tested = 64)	(87.5%) 56/64	O82	**O82:H8**	96	29
		O157	**O157:H7**	6	2
		O177	O177:H19	1	1
		OgN13	OgN13:H19	3	2
			OgN13:H25	1	1
		OgX18	OgX18:H2	4	2
		ONT	ONT:H4	2	1
			ONT:H19	2	2
H (Number of cattle tested = 29)	(31.0%) 9/29	OgX18	OgX18:H8	1	1
		ONT	ONT:H19	4	1
I (Number of cattle tested = 34)	(32.4%) 11/34	O76	O76:H2	1	1
K (Number of cattle tested = 43)	(81.4%) 35/43	O2/O50	O2/O50:H45	13	4
		O22 (1)	O22:H2	1	1
		O153/O178	O153/O178:H19	8	4
		OgN3	OgN3:H19	1	1
		OgX18	OgX18:H2	18	4
M (Number of cattle tested = 34)	(73.5%) 25/34	O2/O50	O2/O50:H45	1	1
		O8	O8:H19	1	1
		O143	O143:H19	1	1
		O26	**O26:H2**	1	1
			**O26:H11**	10	5
		O54	O54:H2	1	1
		O154	**O154:H4**	1	1
N (Number of cattle tested = 88)	19.3% (17/88)	O157	**O157:H7**	8	3
		O171	**O171:H2**	7	3
		O8	**O8:H14**	2	2
		O26	**O26:H11**	1	1
		O76	O76:H14	2	2
		O108	O108:H25	1	1
		ONT	ONT:H2	1	1

Serotypes in** bold** have been previously associated with human disease (diarrhea, bloody diarrhea, hemorrhagic colitis and hemolytic uremic syndrome) (Bettelheim & Goldwater 2019; EFSA BIOHAZ Panel 2013; WHO 1998)

### Occurrence of EPEC in dairy cattle at abattoirs and farms

EPEC was detected in 23.3% (180/771) (95% CI: 20.3%–26.4%) of all dairy cattle sampled at abattoirs and farms (A–N). In addition, EPEC was found in 19.9% (73/367) (95% CI: 15.8%–24.0%) of spent dairy cattle at abattoirs (A–E) and 26.5% (107/404) (95% CI: 22.2%–30.8%) of cattle on farms (F-N). There was a significantly lower likelihood (OR: 0.689; p < 0.05) of EPEC occurrence in spent dairy cattle at abattoirs compared to dairy cattle on farms. The proportion of EPEC positive cattle per abattoir and farm was as follows: A, 24.4% (22/90); B, 15.0% (16/107); C, 36.1% (13/36); D, 14.0% (15/107); E, 26.0% (7/27); F, 24.6% (16/65); G, 14.1% (9/64); H, 62.1% (18/29); I, 11.8% (4/34); J, 0% (0/40); K, 9.3% (4/43); L, 71.4% (5/7) M, 14.7% (5/34) and N, 52.3% (46/88) **(**Table 2**, **Fig. 1**).** Both STEC and EPEC were concurrently detected in 5.6% (43/771) of dairy cattle.

**Table 2:** EPEC occurrence and serotypes in dairy cattle at abattoirs and dairy farms

Abattoirs (A–E) farms (F–N)	Occurrence	O serogroups (N = 16)	Serotypes (N = 19)	Isolates (N = 136)	Number of dairy cattle (N = 771)
A (Number of cattle tested = 90)	(24.4%) 24/90	O26	**O26:H11**	11	6
		O76	**O76:H7**	3	1
		O108	O108:H25	5	1
		O177	**O177:H11**	2	1
		O182	O182:H25	5	1
		OgN9	OgN9:H28	8	2
B (Number of cattle tested = 107)	(15%) 16/107	O10	O10:H-	1	1
			O10:H2	1	1
		O26	**O26:H11**	6	3
			**O26:H21**	1	1
		OgN9	OgN9:H28	5	1
C (Number of cattle tested = 36)	(36.1%) 13/36	O10	O10:H2	8	2
		O26	O26:H2	1	1
		O115	O115:H25	1	1
		O177	O177:H2	1	1
			**O177:H11**	3	1
		OgX18	OgX18:H8	1	1
D (Number of cattle tested = 107)	(14.0%) 15/107	O10	O10:H2	5	1
		O15	**O15:H2**	1	1
		O26	**O26:H11**	5	2
		OgN9	OgN9:H10	1	1
			OgN9:H28	12	4
E (Number of cattle tested = 27)	(26%) 7/27	O10	O10:H2	13	3
H (Number of cattle tested = 29)	(62.1%) 18/29	O10	O10:H25	2	1
		O26	**O26:H11**	1	1
		O49	O49:H10	2	1
		O92	O92:H2	1	1
K (Number of cattle tested = 43)	(9.3%) 4/43	O10	O10:H2	1	1
			O10:H25	11	4
		ONT	ONT:H25	2	2
M (Number of cattle tested = 34)	(14.7%) 5/34	O15	**O15:H2**	1	1
			O15:H-	1	1
		O84	O84:H14	6	3
		ONT	ONT:H-	2	1
N (Number of cattle tested = 88)	(52.3%) 46/88	O2/O50	O2/O50:H10	1	1
		O103	O103:H8	2	2
		O153/O178	O153/O178:H-	1	1
		ONT	ONT:H10	3	3

Serotypes in **bold** have been previously associated with diarrhea in humans (Blanco et al. 2006)

### STEC serotypes

**Fig. 2 a:**
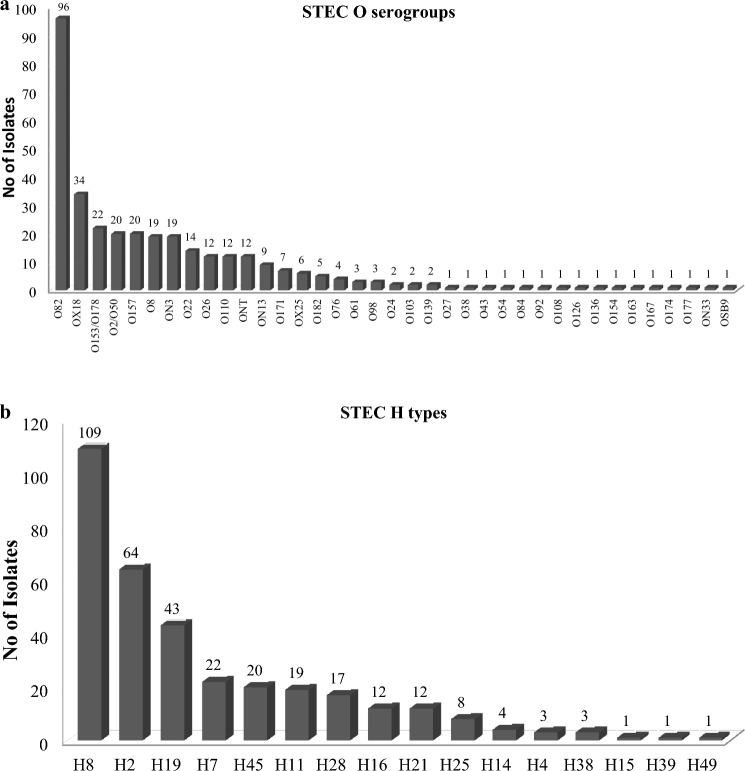
Distribution of O serogroups among dairy cattle STEC isolates. **b** Distribution of H types among dairy cattle STEC isolates

A total of 339 STEC isolates were recovered from 32.3% (105/325) of STEC-positive dairy cattle and 95.6% (324/339) were serotypeable by PCR (Table 1). PCR revealed 53 distinct STEC serotypes including 35 O serogroups and 16 H types while 4.4% (15/339) of the STEC isolates were O-untypable (ONT) (Fig. 2a, b). The O serogroup and H type(s) combinations for STEC can be found in the Supplementary Table S1. Among the 53 STEC serotypes, 52.8% (28/53) were each represented by a single isolate, while the remaining 47.2% (25/53) were represented by more than one isolate (Supplementary Table S3). The highest number of STEC serotypes was observed in abattoir B, with a total of 14 different serotypes, followed by abattoir A, at which recovered STEC isolates belonged to 12 distinct serotypes. Overall, the five most frequent STEC serotypes were O82:H8 (28.3%, 96/339), OgX18:H2 (9.7%, 33/339), O157:H7 (5.9%, 20/339), O2/O50:H45 (5.9%, 20/339) and O153/O178:H19 (5.6%, 19/339). Furthermore, serotypes that belong to “Top 7” STEC serogroups were recovered from 10% (34/339) of all STEC isolates which were serotyped. The following “Top 7” STEC serotypes were identified: O26:H2, 0.3% (1/339); O26:H11, 3.2% (11/339); O103:H8, 0.6% (2/339); and O157:H7, 5.9% (20/339). Top 7″ STEC serotypes were recovered from 4.3% (14/325) of STEC positive animals.

### EPEC serotypes

**Fig. 3 a:**
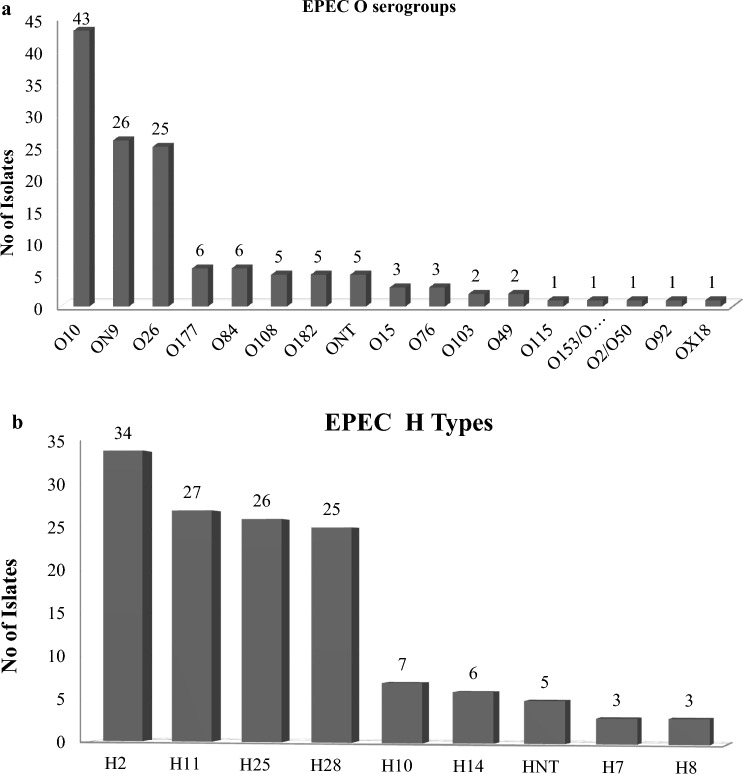
Distribution of O serogroups among dairy cattle EPEC isolates. **b** Distribution of H types among dairy cattle EPEC isolates

Among the 136 EPEC isolates, 92.7% (126/136) were serotypeable by PCR. EPEC serotyping revealed 19 different O:H serotypes including 16 O serogroups and 8 H-types while 5.2% (7/136) isolates were O-nontypeable (ONT) and 2.2% (3/136) were H-nontypable (HNT) (Fig. 3a, b). The 136 EPEC isolates were recovered from 28.9% (52/180) of EPEC-positive dairy cattle. The five most frequent EPEC serotypes were O10:H2 (19.9%, 27/136), ON9:H28 (18.4%, 25/136), O26:H11 (17.6%, 24/136), O10:H25 (9.6%, 13/136) and O84:H14 (4.4%, 6/136). There were 31.6% (6/19) of the EPEC serotypes which were represented by a single isolate, while 68.4% (13/19) were represented by more than one isolate (Supplementary Table S4). The O serogroup and H type (s) combinations for EPEC can be found in Supplementary Table S2.

More than one STEC serotype was isolated from 7.4% (24/325) of STEC positive animals while more than one EPEC serotype was recovered from 2.2% (4/180) of EPEC positive cattle. Furthermore, both STEC and EPEC serotypes were concurrently recovered from 0.8% (6/771) of all animals in the following serotype combinations: STEC O157:H7, STEC O136:H16 and EPEC O153/O178:HNT (1 animal); STEC O8:H21 and EPEC O26:H2 (1 animal); STEC ONT:H19 and EPEC O10:H2 (1 animal); STEC O98:H28 and EPEC O187:H28 (1 animal); STEC O2/O50:H45 and EPEC O10:H2 (1 animal); STEC O153/O178:H19 and EPEC O10:H25 (1 animal).

### Distribution *of major* virulence genes among dairy cattle STEC and EPEC isolates

Both *stx1* and *stx2* were concurrently detected in 196/339 (57.8%) of the isolates, while *stx1* only was detected in 15% (51/339) and *stx2* only was detected in 27.1% (92/339) of isolates.

The *eaeA g*ene was detected in 13.6% (46/339) of the STEC isolates, which corresponded to 6.2% (20/325) of STEC-positive dairy animals. Among the 46 *eaeA*-positive STEC isolates, 69.6% (32/46) were “Top 7” STEC serotypes: O26:H2, 2.1% (1/46); O26:H11, 23.9% (11/46); and O157:H7, 43.5% (20/46). The remaining 30.4% (14/46) of *eaeA*-positive STEC isolates belonged to the following serotypes: O84:H2, O98:H28, O108:H25, O136:H16, O177:H19, O182:H25, OgN3:H19 and OgX18:H8 and were recovered from 2.5% (8/325) of STEC-positive animals.

All the EPEC isolates (100%, 136/136) were *eaeA* positive and *bfpA-*negative while 45.6% (62/136) possessed *hlyA* (Supplementary Table S4).

## Discussion

Current reports on the occurrence of STEC in dairy cattle in South Africa are non-existent. This study investigated the occurrence of STEC and EPEC in dairy cattle on farms and spent dairy animals at abattoirs. STEC was found in 42.2% of the dairy cattle population surveyed. The STEC occurrence observed in this study was higher in comparison to similar reports which reported STEC frequency ranging from 24.7 to 37.5% in different countries including Portugal, Argentina, France and Germany (Ballem et al. 2020; Fernández et al. 2010; Fremaux et al. 2006; Menrath et al. 2010). However, higher STEC occurrence ranging from 62.8 to 82% were reported in studies that investigated STEC in smaller sample sizes of dairy cattle in Brazil and Japan (Cerqueira et al. 1999; Ferreira et al. 2014; Kobayashi et al. 2001). Furthermore, wide variations in the STEC occurrence were also observed among the five abattoirs (18.5–47.2%) and nine dairy farms (14.0–87.5%) which were surveyed. Variations in STEC occurrence among abattoirs and farms may be ascribed to a number risk factors for STEC colonization in cattle including the age of animals, diet, season, health status, and various farm management and hygiene practices (e.g., housing, dry versus wet bedding, pest control, contact with wild animals, manure and slurry disposal). Additional factors which may influence variations in STEC occurrence in cattle are study designs, sampling strategies and sample handling, and laboratory methods and media which are used for enrichment, selection/isolation of STEC.

STEC occurrence was significantly lower in spent cattle at abattoirs (29.9%) in comparison to dairy cattle on farms (53%). The higher frequency of STEC in dairy cattle on farms may be attributed to the younger age of animals on farms in comparison to spent dairy cattle which are older and are sent for slaughter at the end of their productive cycle. Younger animals on farms are easily colonized by STEC because they have a less diverse intestinal microflora (Mir et al. 2016) and an immature immune system which are unable to competitively exclude STEC from the gastrointestinal tract. In contrast, spent dairy cattle are usually older, adult, slaughter age animals which have a more diverse enteric microbiota and mature immune system capable of excluding and counteracting STEC colonization competitively. In addition, previous studies have shown that the prevalence of STEC supershedding is usually greater among younger cattle, which can lead to greater STEC prevalence in cattle (Williams et al. 2015). The presence of STEC supershedders in a particular cattle operation may increase STEC contamination in the environment, which favours faster STEC transmission among animals and subsequent increase in the number of STEC-positive animals.

EPEC was detected in 23.3% of dairy cattle and there was a significantly lower likelihood of EPEC finding in spent cattle at abattoirs (19.9%) compared to dairy cattle on farms (26.5%). So far, only few studies have investigated the presence of EPEC in dairy cattle and have found variable occurrence ranging from 15 to 36% EPEC occurrence in different countries (Eldesoukey et al. 2022; Habets et al. 2020; Singh et al. 2015). While factors that influence EPEC occurrence in dairy cattle populations remain unclear, they could be similar to those that determine STEC occurrence in cattle populations.

It was possible to serotype almost all the STEC isolates (95.6%) by PCR serotyping which revealed 53 STEC serotypes (35 O serogroups and 16 H types). The number of different STEC serotypes identified in this study was higher than reported previously in similar studies on dairy cattle (Fernández et al. 2010; Irino et al. 2005; Menrath et al. 2010). This could be attributed to the use of PCR serotyping (Banjo et al. 2018; DebRoy et al. 2018; Iguchi et al. 2015, 2016, 2020; Ludwig et al. 2020; Singh et al. 2015) instead of traditional serotyping (Ørskov & Ørskov 1984). PCR serotyping has shown high sensitivity and specificity for identifying *E. coli* serotypes (Malahlela et al. 2022), particularly for isolates which are O nontypeable and/or H nontypeable (ONT/HNT) by traditional serotyping (Banjo et al. 2018; DebRoy et al. 2018; Iguchi et al. 2015, 2016, 2020; Ludwig et al. 2020; Singh et al. 2015). With PCR serotyping it is possible to identify *E. coli* strains that carry but are unable to express genes encoding somatic O and flagellar H antigens (O rough and nonmotile).

Only 10% of dairy cattle STEC isolates belonged to “Top 7” STEC serotypes, consistent with studies which have also observed low occurrence of the “Top 7” STEC, ranging from 2.4 to 13.3% in STEC from dairy cattle (Ballem et al. 2020; Bibbal et al. 2015; Cerqueira et al. 1999; Fernández et al. 2010). The following “Top 7” STEC serotypes were observed in this study: STEC O157:H7, STEC O26:H11/H2 and STEC O103:H8. While both O157:H7 and O26:H11 are considered the two most clinically relevant STEC serotypes globally including South Africa (EFSA 2013; Bettelheim and Goldwater 2019; Karama et al. 2019a; Smith et al. 2019), the clinical importance of STEC O26:H2 and STEC O103:H8 remains unclear, as both serotypes have been rarely reported in human disease (Baba et al. 2019; Bettelheim & Goldwater 2019). Furthermore, STEC O157:H7 and O26:H11 are responsible for most human STEC outbreaks and common in severe disease, and the most frequent serotypes in human STEC foodborne outbreaks linked to consumption of contaminated dairy products (Farrokh et al. 2013; Hussein & Sakuma 2005b). In addition, 46% of STEC isolates belonged to non-Top 7 serotypes which have been associated with human infections around the world including South Africa (STEC O22:H16 and O8:H19) (Karama et al. 2019a) (STEC O8:H14, O8:H21, O22:H8, O22:H11, O82:H8, O84:H2, O110:H19, O153/O178:H7, O154:H4, O171:H2 and O174:H28) (Bettelheim & Goldwater 2019; WHO 1998). The isolation of STEC serotypes which have been previously implicated in human disease supports the role of dairy cattle as a reservoir and potential source of STEC in South Africa.

Similar to STEC, almost all (92.7%) of EPEC isolates were serotypeable by PCR which revealed 19 different serotypes including EPEC O15:H2, O26:H11, O76:H7 and O177:H11 which have been previously implicated in human disease (Blanco et al. 2006). Of particular interest, was the isolation of aEPEC O26:H11 which is one of the frequent and clinically relevant EPEC serotype in infantile diarrheae, worldwide (Croxen et al. 2013; Durso et al. 2005).

Furthermore, all EPEC isolates were classified as atypical EPEC (aEPEC) *bfpA-*negative), in agreement with other studies which have mainly detected strains from dairy cattle (Auvray et al. 2023; Bibbal et al. 2015; Singh et al. 2015). While most human EPEC infections have been linked with typical EPEC (*bfpA*-positive), the association of aEPEC with infantile diarrheae remains controversial (Hernandes et al. 2009). However, some reports found various serotypes of aEPEC strains in children with acute diarrhea, in various age groups and patients with AIDS (reviewed by Hernandes et al. 2009).

Virulotyping of 339 STEC isolates revealed that *stx2*-positive STEC isolates were more frequent than *stx1*-positive among dairy STEC isolates**.** This finding is in agreement with previous studies that characterized STEC from dairy cattle and reported higher frequency of *stx2* than *stx1* (Ballem et al. 2020; Bibbal et al. 2015; Dong et al. 2017; Fernández et al. 2010; Venegas-Vargas et al. 2016). However, other studies showed that *stx1* was more frequent than *stx2* in dairy cattle (Cerqueira et al. 1999; Ferreira et al. 2014; Singh et al. 2015). Previous studies have shown that *stx2*-carrying STEC isolates are more frequent in human STEC disease, particularly severe human STEC infections characterized by bloody diarrhea, hemorrhagic colitis and the hemolytic uremic syndrome, a complication commonly associated with renal failure and dialysis, worldwide (Boerlin et al. 1999; Friedrich et al. 2002; Obrig & Karpman 2012).

While most dairy cattle STEC were *eaeA*-negative, *eaeA* was detected in only 13.6% of all STEC isolates. The *eaeA* gene was mainly observed among “Top 7” STEC O26:H2, O26:H11 and O157:H7 isolates and non-Top 7 including O84:H2, O98:H28, O108:H25, O136:H16, O182:H25, OgN3:H19 and OgX18:H8 (Bettelheim & Goldwater 2019; EFSA BIOHAZ Panel 2013; WHO 1998). The low occurrence of *eaeA* in dairy STEC isolates is in agreement with previous studies which reported low occurrence of *eaeA* among dairy cattle STEC (Cobbold & Desmarchelier 2001; Fernández et al. 2010; Irino et al. 2005). Considering the clinical significance and strong association of *eaeA* with highly virulent STEC strains (EFSA BIOHAZ Panel., 2020; Ethelberg et al. 2004), the high occurrence of *eaeA* among STEC isolates suggests that these *eaeA*-positive dairy cattle STEC isolates are of high risk to humans and will need to be closely monitored during STEC epidemiological surveillance.

Most dairy cattle STEC isolates (69.9%) and a considerable number of EPEC (46.5%) were *hlyA*-positive. While the significance of *hlyA* in STEC and EPEC virulence is not clear, some studies have suggested that *hlyA* is a potential EPEC and STEC virulence factor (Aldick et al. 2009; Schwidder et al. 2019). In addition, the presence of *hlyA* in STEC strains was associated with severe human STEC disease including hemorrhagic colitis and hemolytic uremic syndrome (Schmidt & Karch 1996). Furthermore, previous reports (Aldick et al. 2007; Bielaszewska et al. 2013) have suggested that possession and production of *hlyA* was associated with damage of the endothelial barrier of the vascular system through pore-formation, subsequent cell lysis and apoptosis, thereby contributing to the development of HUS.

The findings from this study demonstrated that dairy cattle in South Africa are a reservoir of STEC and EPEC. The detection of virulent STEC and EPEC serotypes which have been previously incriminated in human disease around the world, including South Africa, underscores the significance of dairy cattle as reservoir and potential source of clinically relevant STEC and EPEC. The STEC isolates recovered in this study will need to be further characterized to ascertain the full virulence potential of dairy cattle STEC and EPEC for humans. In addition, molecular characterization studies comparing cattle STEC and EPEC with human isolates will have to be carried to determine the role played by cattle in the transmission and causation of STEC and EPEC infections in humans in South Africa.

## Supplementary Information

Below is the link to the electronic supplementary material.

Supplementary file1 (DOCX 34 KB)

## Data Availability

No datasets were generated or analysed during the current study.
