# Supplementary material for: Occurrence, serotypes and virulence characteristics of Shiga toxin-producing and Enteropathogenic Escherichia coli isolates from dairy cattle in South Africa
Source: World J Microbiol Biotechnol. 2024 Aug 13;40(10):299. doi: 10.1007/s11274-024-04104-w (PMC11319423; doi:10.1007/s11274-024-04104-w)
Supplement: Supplementary file 1 — Supplementary file1 (DOCX 34 KB) [file 11274_2024_4104_MOESM1_ESM.docx]

**Supplementary Materials**

**Table S1:** Association between O group and H type(s) among dairy cattle STEC isolates

| **O-Group** | **Associated H-type** | | | | | | |
| --- | --- | --- | --- | --- | --- | --- | --- |
| O2/O50 | H45 (20) |  |  |  |  |  |  |
| O8 | H2 (1) | H8 (3) | H14 (2) | H19 (3) | H21 (9) | H28 (1) | H38 (1) |
| O22 | H8 (5) | H16 (8) |  |  |  |  |  |
| O24 | H38 (2) |  |  |  |  |  |  |
| O26 | H2 (1) | H11 (11) |  |  |  |  |  |
| O27 | H21 (1) |  |  |  |  |  |  |
| O38 | H8 (1) |  |  |  |  |  |  |
| O43 | H8 (1) |  |  |  |  |  |  |
| O54 | H2 (1) |  |  |  |  |  |  |
| O61 | H16 (3) |  |  |  |  |  |  |
| O76 | H2 (2) | H14 (2) |  |  |  |  |  |
| O82 | H8 (96) |  |  |  |  |  |  |
| O84 | H2 (1) |  |  |  |  |  |  |
| O92 | H28 (1) |  |  |  |  |  |  |
| O98 | H28 (3) |  |  |  |  |  |  |
| O103 | H8 (2) |  |  |  |  |  |  |
| O108 | H2 (1) |  |  |  |  |  |  |
| O110 | H19 (2) | H28 (10) |  |  |  |  |  |
| O136 | H16 (1) |  |  |  |  |  |  |
| O139 | H8 (1) | H15 (1) |  |  |  |  |  |
| O143 | H19 (1) |  |  |  |  |  |  |
| O153/O178 | H7 (1) | H19 (19) | H21 (1) | H49 (1) |  |  |  |
| O154 | H4 (1) |  |  |  |  |  |  |
| O157 | H7 (20) |  |  |  |  |  |  |
| O163 | H21 (1) |  |  |  |  |  |  |
| O167 | H25 (1) |  |  |  |  |  |  |
| O171 | H2 (7) |  |  |  |  |  |  |
| O174 | H28 (1) |  |  |  |  |  |  |
| O177 | H19 (1) |  |  |  |  |  |  |
| O182 | H25 (5) |  |  |  |  |  |  |
| OgN3 | H2 (18) | H19 (1) |  |  |  |  |  |
| OgN13 | H19 (8) | H25(1) |  |  |  |  |  |
| OgN33 | H19 (1) |  |  |  |  |  |  |
| OgX18 | H2 (33) | H8 (1) |  |  |  |  |  |
| OgX25 | H11 (5) | H28 (1) |  |  |  |  |  |
| ONT | H2 (1) | H4 (2) | H7 (1) | H8 (2) | H19 (8) | H39 (1) |  |

Numbers in parentheses represent the number of dairy cattle STEC isolates

**Table S2:** Association between O group and H type(s) among dairy cattle EPEC isolates

| **O-Group** | **Associated H-type** | | |
| --- | --- | --- | --- |
| O2/O50 | H10 (1) |  |  |
| O10 | H2 (27) | H25 (13) | H- (1) |
| O15 | H2 (2) | H- (1) |  |
| O26 | H2 (1) | H11 (24) |  |
| O49 | H10 (2) |  |  |
| O76 | H7 (3) |  |  |
| O84 | H14 (6) |  |  |
| O92 | H2 (1) |  |  |
| O103 | H8 (2) |  |  |
| O108 | H25 (5) |  |  |
| O115 | H25 (1) |  |  |
| O153/O178 | H-(1) |  |  |
| O177 | H2 (4) | H11(2) |  |
| O182 | H25 (5) |  |  |
| OgN9 | H10 (1) | H28 (25) |  |
| OgX18 | H8 (1) |  |  |
| ONT | H10 (3) | H25 (2) | H-(2) |

Numbers in parentheses represent the number of dairy cattle EPEC isolates

**Table S3.** Distribution of major virulence genes among dairy cattle STEC serotypes

| **Serotype** | ***stx1*** | ***stx2*** | ***eaeA*** | ***hlyA*** | **No of Isolates** |
| --- | --- | --- | --- | --- | --- |
| O2/O50:H45 | + | - | - | - | 1 |
| O2/O50:H45 | + | - | - | + | 19 |
| **O8:H8** | - | + | - | + | 2 |
| **O8:H14** | + | - | - | - | 2 |
| **O8:H19** | - | + | - | + | 1 |
| **O8:H19** | + | + | - | + | 2 |
| **O8:H21** | - | + | - | - | 9 |
| O8:H28 | - | + | - | + | 1 |
| O8:H38 | - | + | - | + | 1 |
| O22:H2 | + | + | - | - | 1 |
| **O22:H8** | - | + | - | - | 5 |
| O22:H16 | - | + | - | - | 8 |
| O24:H38 | + | + | - | - | 1 |
| O24:H38 | + | + | - | + | 1 |
| **O26:H2** | + | + | + | + | 1 |
| **O26:H11** | + | - | + | + | 11 |
| O27:H21 | + | - | - | - | 1 |
| O38:H8 | - | + | - | - | 1 |
| O43:H8 | + | - | - | - | 1 |
| O54:H2 | + | + | - | + | 1 |
| O61:H16 | - | + | - | + | 3 |
| O76:H2 | + | + | - | + | 2 |
| O76:H14 | + | - | - | - | 2 |
| **O82:H8** | + | + | - | - | 31 |
| **O82:H8** | + | + | - | + | 65 |
| O84:H2 | + | - | + | + | 1 |
| O92:H28 | + | + | - | - | 1 |
| O98:H28 | + | - | + | + | 3 |
| O103:H8 | - | + | - | - | 2 |
| O108:H2 | + | - | + | + | 1 |
| **O110:H19** | - | + | - | - | 2 |
| O110:H28 | - | + | - | - | 10 |
| O136:H16 | + | + | + | + | 1 |
| O139:H8 | + | + | - | + | 1 |
| O139:H15 | + | + | - | + | 1 |

**Table S3.** *Cont.*

| **Serotype** | ***stx1*** | ***stx2*** | ***eaeA*** | ***hlyA*** | **No of Isolates** |
| --- | --- | --- | --- | --- | --- |
| O143:H19 | + | + | - | + | 1 |
| O153/O178:H7 | - | + | - | - | 1 |
| O153/O178:H19 | - | + | - | - | 4 |
| O153/O178:H19 | + | + | - | - | 15 |
| O153/O178:H21 | - | + | - | - | 1 |
| O153/O178:H49 | + | + | - | - | 1 |
| **O154:H4** | + | - | - | - | 1 |
| **O157:H7** | - | + | + | + | 20 |
| O163:H21 | + | - | - | - | 1 |
| O167:H25 | + | + | - | + | 1 |
| **O171:H2** | + | + | - | + | 7 |
| **O174:H28** | + | + | - | + | 1 |
| O177:H19 | - | + | + | + | 1 |
| O182:H25 | + | - | + | + | 5 |
| OgN13:H19 | - | + | - | + | 8 |
| OgN13:H25 | - | + | - | + | 1 |
| OgN3:H2 | + | + | - | + | 18 |
| OgN3:H19 | + | - | + | + | 1 |
| OgN33:H19 | - | + | - | - | 1 |
| OgX18:H8 | + | + | + | + | 1 |
| OgX18:H2 | + | + | - | + | 33 |
| OgX25:H28 | - | + | - | + | 1 |
| OgX25:H11 | - | + | - | + | 5 |
| ONT:H2 | + | - | - | - | 1 |
| ONT:H4 | + | + | - | + | 2 |
| ONT:H7 | - | + | - | - | 1 |
| ONT:H8 | - | + | - | - | 1 |
| ONT:H8 | + | + | - | - | 1 |
| ONT:H19 | + | + | + | - | 1 |
| ONT:H19 | + | + | - | + | 5 |
| ONT:H19 | - | + | - | - | 2 |
| ONT:H39 | + | + | - | + | 1 |
| **Total** | 247 | 288 | 46 | 237 | 339 |
| **% Positive** | 72.9 | 85.7 | 13.6 | 69.9 |  |

Serotypes in **bold** have been previously associated with human disease (Diarrhea, Bloody diarrhea, Hemorrhagic colitis and Hemolytic uremic syndrome) (WHO, 1998; Bettelheim and Goldwater, 2019)

**Table S4.** Distribution of virulence genes among dairy cattle EPEC serotypes

| **Serotype** | ***eaeA*** | ***hlyA*** | ***bfp*** | **No of Isolates** |
| --- | --- | --- | --- | --- |
| O2/O50:H10 | + | - | - | 1 |
| O10:H2 | + | - | - | 27 |
| O10:H2 | + | + | - | 1 |
| O10:H25 | + | - | - | 3 |
| O10:H25 | + | + | - | 10 |
| O10:HNT | + | - | - | 1 |
| **O15:H2** | + | - | - | 2 |
| O15:HNT | + | + | - | 1 |
| O26:H2 | + | - | - | 1 |
| **O26:H11** | + | - | - | 24 |
| O49:H10 | + | + | - | 2 |
| **O76:H7** | + | + | - | 3 |
| O84:H14 | + | - | - | 6 |
| O92:H2 | + | - | - | 1 |
| O103:H8 | + | - | - | 2 |
| O108:H25 | + | + | - | 5 |
| O115:H25 | + | + | - | 1 |
| O153/O178:HNT | + | - | - | 1 |
| O177:H2 | + | + | - | 4 |
| **O177:H11** | + | + | - | 2 |
| O182:H25 | + | + | - | 5 |
| OgN9:H10 | + | + | - | 1 |
| OgN9:H28 | + | - | - | 2 |
| OgN9:H28 | + | + | - | 23 |
| OgX18:H8 | + | + | - | 1 |
| ONT:H10 | + | - | - | 3 |
| ONT:H25 | + | + | - | 2 |
| ONT:HNT | + | + | - | 2 |
| **Total** | 136 | 62 | 0 | 136 |
| **% Positive** | 100 | 45.6 | 0 |  |

Serotypes in **bold** have been previously associated with diarrhea in humans (Blanco *et al.*, 2006)
